# The status of postapproval monitoring operation by the Institutional Animal Care and Use Committee in Korea

**DOI:** 10.1186/s42826-025-00258-2

**Published:** 2025-10-15

**Authors:** Jimin Lee, Na Ahn, Sangho Roh

**Affiliations:** 1 Institute of Environmental Protection & Safety, Seoul National University, Seoul, Korea; 2 College of Medicine, Kyung Hee University, Seoul, Korea; 3 School of Dentistry and Dental Research Institute, Seoul National University, Seoul, Korea

**Keywords:** Animal ethics, Animal welfare, IACUC, Postapproval monitoring

## Abstract

**Background:**

Postapproval monitoring (PAM) is a critical component of Institutional Animal Care and Use Committee (IACUC) oversight, ensuring compliance with approved protocols and ethical animal research practices. While the PAM is not explicitly mandated under U.S. federal regulations, it has been widely recognized as an essential mechanism for verifying adherence to animal welfare standards. In contrast, Korea has formally integrated the PAM into its legal framework, making it a mandatory function of IACUCs.

**Results:**

This study examines the implementation and perception of the PAM in Korean institutions through a survey of relevant professionals. These findings indicate that while awareness of the PAM is high, challenges such as limited manpower and institutional support hinder its effective execution. Additionally, the pandemic highlighted the potential for online remote monitoring as a supplemental method, although concerns remain regarding its effectiveness in assessing real-time animal welfare conditions. The study suggests that a hybrid PAM model, that combines onsite and remote monitoring, could improve oversight efficiency while addressing resource constraints. Strengthening administrative support, increasing professional staffing, and enhancing researcher training are crucial steps for optimizing PAM operations in Korea.

**Conclusions:**

These insights contribute to the broader discourse on the evolution of animal research oversight and the need for adaptable monitoring strategies in diverse regulatory environments.

**Supplementary Information:**

The online version contains supplementary material available at 10.1186/s42826-025-00258-2.

## Background

In institutions that use animals in research or education, a committee called the Institutional Animal Care and Use Committee (IACUC) oversees animal welfare standards and is responsible for ensuring compliance with applicable governmental regulations, policies, and guidelines. IACUCs play a critical role in animal care and use programs, ensuring that institutions that use animals in research and training do so responsibly and humanely [1]. In the United States, IACUCs are typically assigned at least four core functions: inspecting animal facilities and animal use areas; reviewing the institution’s program for animal care and use; investigating concerns involving animal care and use; and reviewing animal protocols [2]. To review animal protocols in Korea, for example, administrators review protocol paperwork first, and then assign the protocols to IACUC members for review. Once a reviewer has examined the protocol, the committee provides one of four outcomes: (1) approved, (2) conditionally approved with a request for revision, (3) re-review after revision, or (4) rejected [3]. In addition to a review of the protocol, oversight of animal activities is one of the most important responsibilities of the IACUC. This ongoing monitoring and reporting of animal research activities, referred to as postapproval monitoring (PAM), is needed, as stated in the laws governing the care and use of animals in research, training, and testing activities [4, 5]. The definition of the PAM proposed by Collins in 2008 [6] is as follows. “PAM can be briefly defined as any effort focused on determining what happens to animals after IACUC approval has been granted for their use in research, teaching, or testing”.

There are no explicit federal regulations or guidelines specifying a PAM program in the United States [6]. Therefore, the PAM is not considered a mandated role of the IACUC according to U.S. federal laws, regulations, and guidelines. However, since the late 1990s, the fundamental concept of the PAM was introduced, and since its definition in 2008, the PAM has been recognized as a behavioral approach for fulfilling the role of the IACUC. It is considered an important method to ensure that researchers meet the requirements set by the IACUC. As an accrediting organization, the Association for Assessment and Accreditation of Laboratory Animal Care (AAALAC) International emphasizes the importance of having a mechanism in place to review that animal research procedures are conducted by approved protocols, even in the absence of specific laws or regulations requiring PAM [7]. As an institution’s animal protocol oversight regulator, the IACUC can use a PAM to ensure that it meets its regulatory requirements [8]. However, PAM programs that are strictly compliance-focused may not be fit for animal research activities by license holders. This is because, in such a scenario, an IACUC that is geared toward animal facility compliance could cite minor facility noncompliance as posing a disproportionate issue to animal welfare for license-holders using the facility, leading to financial penalties, poor-quality research results, loss of research time and negative publicity to the institution and license holder for their use of the facility under inspection [9]. Researchers may perceive the PAM as an additional regulatory burden as regulations expand. However, allowing research institutions to design customized PAM programs helps minimize unnecessary burdens while ensuring compliance. Moreover, an effective PAM program can prevent potential noncompliance issues before they become major concerns, fostering a more flexible research environment [4]. Implementing a PAM system helps ensure compliance with approved protocols by monitoring animals, administering analgesics as prescribed, and maintaining proper documentation [8].

According to the Rules of AAALAC, access to facilities and information is essential to assess all aspects of husbandry, sanitation, animal well-being, and safety procedures. Institutions subject to accreditation are required to establish a system that ensures animal research procedures are conducted in accordance with approved protocols or study plans, following the recommendations for a PAM program as outlined in The Guide [7]. An analysis of the PAM methods and recommended examples proposed in The Guide reveals that they can be broadly categorized into observation-based direct activities and document-based indirect activities (Table A1). This distinction serves as a critical criterion for resource allocation and prioritization such as personnel, time, and budget when designing a PAM strategy [2]. Observation-based direct activities require personnel to be physically present on-site, demand more time, and necessitate specialized veterinary or technical expertise. In contrast, document-based activities can be conducted remotely in an office setting and generally require fewer personnel and less time.

In Korea, PAM activities typically focus on the inspection of animal facilities and their operations, and not the program of animal care and use. This situation is now changing. The revised Animal Protection Act establishes the PAM as an institutional obligation. The primary activity of PAM which involves onsite monitoring is to ensure that approved animal protocols are complied with, verifying factors such as the number of animals used, and that only the experiments that are outlined in the protocols are conducted [10, 11]. The PAM in Korea was explicitly designated as an important role of the IACUC, marking the world’s first such acknowledgment. According to a recent Korean survey on animal rights, 3.13 million households raise pets, and the proportion of people who consider animals as families is increasing. Although animal testing is necessary (78%) and ethically justified (52%), 48% of people say that the welfare level of experimental animals is low [12]. This reflects that our society has a dual attitude toward animal rights, including experimental animals, but it has become an inevitable task to manage animal experiments more strictly. In addition, as younger generations of animal researchers are increasingly aware of animal rights, monitoring animal experiments or laboratory animal management, or PAM, has become one of the most important activities in IACUC operations to care for not only animals but also researchers themselves [13]. Several cases related to the supply of laboratory animals in Korea in recent years highlight the importance of not only the approval of the animal protocol but also the activities to monitor it afterward [14]. These examples include the following. (1) Obtaining foster and oocyte donor dogs from a dog meat farm to carry out their university-affiliated dog cloning program [15] (2) Purchasing dogs for education and training in a veterinary school from an unclear source: the presumed source of dogs was a local dog meat shop, which could not be a registered animal supplier [16] (3), Animal abuse after conducting unnecessary experiments on cats and euthanizing them without proper anesthesia [17].

## Methods

The methodological foundation of this study was based upon a qualitative approach, employing questionnaires and semi-structured interviews. Ethical approval for the study was secured from the Institutional Review Board of the Seoul National University School of Dentistry, with the approval number: S-D20240006. We utilized the Google survey system as our primary tool for data collection. Before participating, all respondents were briefed on the objectives of the study, ensuring their informed consent and their understanding of their rights as study participants.

### Theoretical sampling of respondents

Theoretical sampling is an approach to purposeful sampling designed around categories that we devised for this study, which are anchored in theoretical considerations. Thirty individuals from each institution were surveyed via purposeful sampling. We selected our primary respondents, the animal facility managers and IACUC administrators (secretaries), on the basis of diverse institution types such as universities, private companies, and hospitals to ensure broad representation in our group of respondents. Furthermore, researchers holding positions as laboratory directors or managers, who are responsible for submitting animal protocols, were also selected from the institutions.

### Questionnaire composition of the survey

The questionnaire was composed of items necessary for understanding the operation of the PAM of the IACUC. Each of these parts consists of multiple-choice questions and narrative (descriptive) questions, which are considered short written interviews. In some institutions, the IACUC administrator also serves as an animal facility manager.

### Data analyses

Survey data were extracted from the respondents’ responses. Once the survey results from multiple-choice questions were collated, for narrative (descriptive) questions, all three authors independently grouped answers from descriptive questions with similarities, and the results were determined after discussion. This allowed us to structure the data and determine response rates for each specific item. On the basis of this consolidated information, we identified critical challenges and considerations for the PAM operation. This led us to recommendations to address needs.

## Results

### Awareness of the PAM

As a result of a survey of awareness of the PAM (Table 1), all 30 respondents responded that they had heard of the PAM, and the majority (90%) said that they understood the purpose of the legal provisions to some extent, but some respondents (10%) said that they had a low or insufficient understanding. This suggests that the PAM is already well known among respondents, although some need more understanding. In addition, the majority of respondents (93.3%) recognized that the PAM is a necessary procedure and that they believe that the PAM contributes to systemization and strengthening the ethics of animal experiments, indicating that they highly understand the positive effects of the PAM. Although many respondents (73.3%) reported that the PAM was implemented in accordance with the purpose of the Animal Protection Act, some suggested neutral (23.3%) or negative (3.3%) results.

**Table 1:** Awareness of postapproval monitoring (PAM)

	Highly agree (%)	Agree (%)	Neutral (%)	Not agree (%)	Highly not agree (%)
Do you fully understand the purpose of the PAM in the Animal Protection Act?	18 (60)	9 (30)	2 (6.7)	-	1 (3.3)
Do you think PAM is a necessary procedure?	23 (76.7)	5 (16.7)	2 (6.7)	-	-
Do you think PAM helps researchers to conduct animal experiments more systematically and ethically?	14 (46.7)	14 (46.7)	2 (6.7)	-	-
Do you think PAM operations are being properly implemented in accordance with the purpose of the Animal Protection Act in your institution?	11 (36.7))	11 (36.7)	7 (23.3)	1 (3.3)	-

### Implementation of the PAM

A survey of the implementation of the PAM (Table 2) revealed that 27 respondents had experience in implementing the PAM at their institution. Among them, more than half responded that they had implemented it during the last three years, and another 10 respondents said that they had implemented it for the first time last year, indicating that the revised Animal Protection Act last year affected the spread of the PAM. The most common annual frequency of implementation was once a year (40%), and 30% and 20% were implemented twice a year or more than three times a year, respectively (Table 2).

**Table 2:** Implementation of postapproval monitoring (PAM)

	Do not implement	For the first time last year			During the last three years
Is the PAM implemented by your institution?	3 (10.0%)	10 (33.3%)			17 (66.7%)
	Do not implement	Once a year	Twice a year		More than 3 times a year
How many times is the PAM implemented by your institution?	3 (10.0%)	12 (40.0%)	9 (30.0%)		6 (20.0%)

The answers to descriptive questions (multiple answers are possible) asking why a PAM is needed could be grouped into five answers (Table 3). Among them, the most common answer was ‘Verify that it is performed as the protocol applied’ (73.3%) followed by ‘Compliance with Animal Welfare and Animal Ethics’ (26.7%). In addition, the majority of the respondents answered the descriptive question of what benefits the institution had while conducting the PAM, saying, ‘Compliance with experiments as per protocol’ (36.7%) and ‘Understanding relevant laws, animal welfare and veterinary treatment’ (30.0%) (Table 4). Tables 3 and 4 show that the respondents have a good understanding of the original purpose of the the PAM.

**Table 3:** The reason postapproval monitoring should be performed

Reasons*	Number (%) of respondents
**Verify that it is performed as the protocol approved**	**22 (73.3)**
**Compliance with animal welfare and animal ethics**	**8 (26.7)**
Improve understanding of animal experiments	4 (13.3)
Because it is the basic role of the committee	3 (10.0)
Necessary but difficult to apply to all animal experiments	1 (3.3)

*Requested descriptive answers, and 38 answers from 30 respondents

**Table 4:** Benefits from postapproval monitoring operation

Benefits you think the most*	Number %) of respondents
**Compliance with experiments as per protocol**	**11 (36.7)**
**Understanding relevant laws**,** animal welfare and veterinary treatment**	**9 (30.0)**
Careful from animal protocol writing	3 (10.0)
Identifying research reliability and improvements	2 (6.7)

*Requested descriptive answers, and collected 25 answers from 30 respondents

### Selecting the PAM targets

For the PAM, it was most common to select targets by the IACUC (70.0%). The second method involved selecting targets by management teams such as attending veterinarians (AVs), managers, staff, etc. (43.3%). In addition, four respondents (13.3%) said they would select a target through a researcher’s report, and two (6.7%) responded that they would check all animal experiments, indicating that in most cases, the committee or management team chose the target (Table 5).

**Table 5:** Selecting postapproval monitoring (PAM) targets

Selecting the PAM target (all that applies)*	No. of respondents (%)
**By the committee**	**21 (70.0)**
**By the management team (attending vet**,** manager**,** staff)**	**13 (43.3)**
Through a researcher’s report	4 (13.3)
All animal experiments	2 (6.7)
PAM not implement	1 (3.3)
Don’t know	1 (3.3)

*Multiple responses allowed from 30 respondents

### Implementation of online remote monitoring (non-face-to-face)

The most common the PAM during the non-pandemic period was onsite monitoring (86.7%), followed by self-check document review (26.7%) and online remote monitoring (6.7%). Even during the pandemic, onsite monitoring was still the highest at 43.3%, but the proportion decreased remarkably compared with that in the non-pandemic period; instead, the proportion of non-face-to-face remote monitoring (23.3%) increased. Interestingly, the proportion of self-check document reviews (20.0%) during the pandemic did not differ significantly from the results of the non-pandemic period. Online remote monitoring may be preferred as an alternative to self-check document review as a way to reduce face-to-face contact during a pandemic (Table 6).

**Table 6:** Changes in postapproval monitoring (PAM) due to pandemic

Type of monitoring (all that applies)*	Number of institutions implementing the PAM during the	
	Pandemic period (%)	Non-pandemic period (%)
On-site monitoring	13 (43.3)	26 (86.7)
Self-check document review	6 (20.0)	8 (26.7)
Online remote monitoring	7 (23.3)	2 (6.7)

*Multiple responses allowed from 30 respondents

**Fig. 1:**
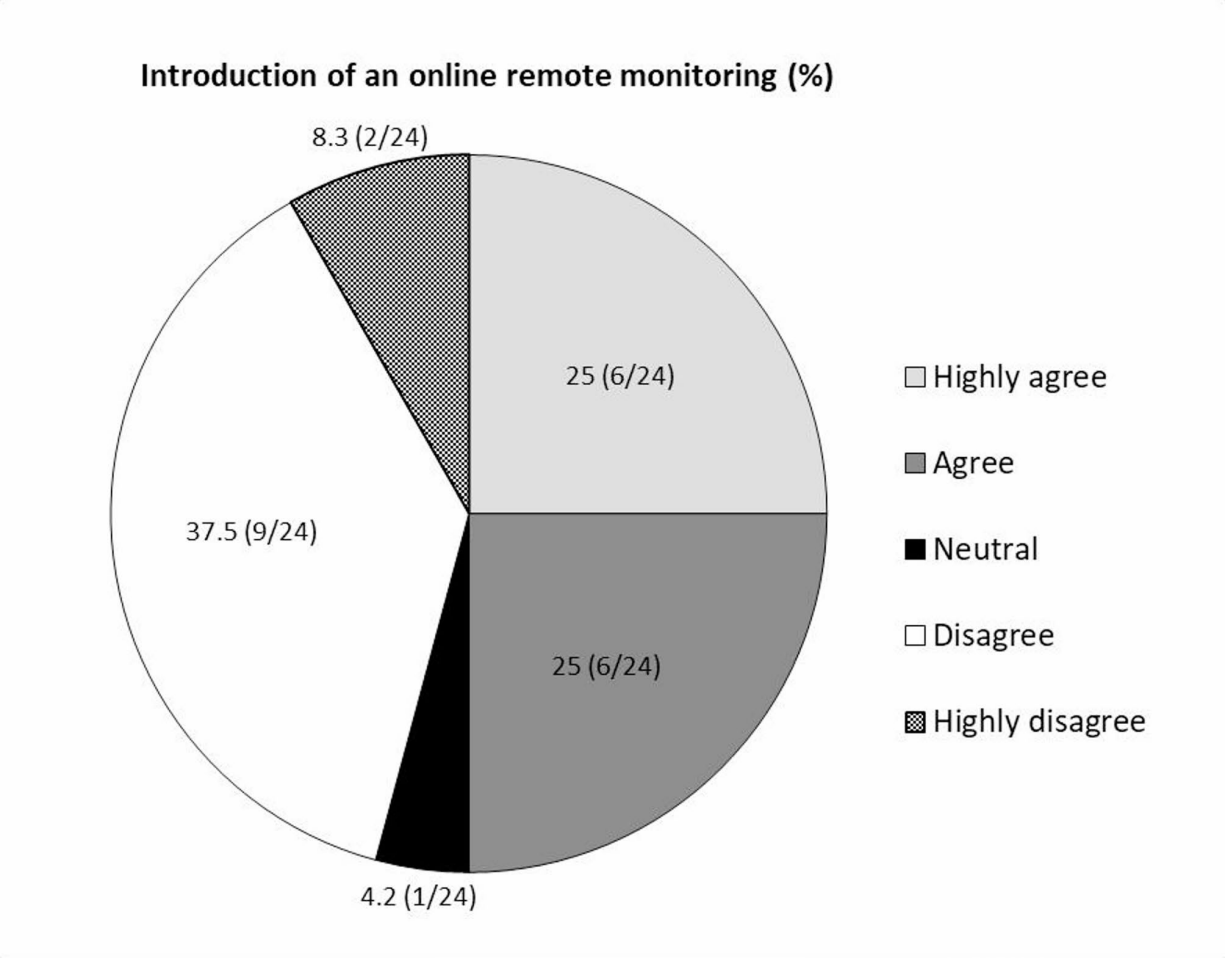
Views on online remote monitoring for postapproval monitoring. When asked about the intention to introduce non-face-to-face online remote monitoring, 50% of the effective respondents positively evaluated the introduction of online remote monitoring (25% highly agree and 25% agree), whereas 50% of the remaining respondents were still reluctant to introduce online remote monitoring (37.5% highly disagree, 8.3% highly disagree, and 4.2% neutral)

Although onsite monitoring is, of course, a main form of the PAM, online remote monitoring can be an alternative in disaster situations such as pandemics. When asked about the intention to introduce the non-face-to-face online remote monitoring, 50% of the effective respondents positively evaluated the introduction of online remote monitoring (25% highly agree and 25% agree), indicating that they have the potential to replace or supplement existing onsite monitoring (Fig. 1). However, since 50% of the remaining respondents were still reluctant to use online remote monitoring (37.5% highly disagree, 8.3% highly disagree, and 4.2% neutral), there still seems to be a great concern about remote monitoring.

The answers to the descriptive questions asking the participants whether they agreed or disagreed with online remote monitoring are shown in Table 7. There are various opinions on the main reasons for agreeing with online remote monitoring, such as ‘Easy to include the number of inspections’ (3 of 11) and ‘Save time and manpower input’ (3 of 11), whereas the reasons for disagree were overwhelmingly due to ‘Difficulty knowing the actual situation of the animal facility’ (9 of 14). This suggests that even if online remote monitoring is actively applied in the future, much supplementation is needed.

**Table 7:** The reasons for agreeing or disagree with online remote monitoring*

Agree	Number of respondents
Easy to increase the number of inspections	3
Save time and manpower input	3
Monitor whenever you want	2
Useful in emergency situations such as pandemic	1
Useful when you have multiple animal facilities	1
Enough to achieve the goal	1
Disagree	Number of respondents
**Difficulty knowing the actual situation of the animal facility**	**9**
Difficult to communicate with researchers, form consensus, and provide immediate feedback	4
On-site monitoring is in line with the purpose of the monitoring	1

*Request descriptive answers

### Difficulties in PAM operation

The respondents answered that ‘Lack of manpower’ was the greatest difficulty in PAM operation (50%), followed by ‘Lack of awareness or cooperation of researchers’ (46.7%) and ‘Lack of awareness or administrative support by institutions’ (26.7%) (Table 8). This result shows that manpower support and lack of awareness/cooperation among researchers are the greatest challenges to be solved in PAM operation.

**Table 8:** Difficulties in postapproval monitoring operation

Reason of difficulty (all that applies)*	Number of respondents (% from 30 respondents)
**Lack of manpower**	**15 (50.0)**
**Lack of awareness or cooperation of researchers**	**14 (46.7)**
Lack of awareness or administrative support by institutions	8 (26.7)
Lack of awareness of the committee members	2 (6.7)
N/A	2 (6.7)

*Multiple responses allowed from 30 respondents

Finally, as a result of inquiring opinions and suggestions to survey participants, various answers were obtained, including ‘Understanding and regular training of relevant laws’ (5 of 11) and ‘Recruitment of professional personnel including AVs’ (3 of 11) (Table 9). For seamless operation of the PAM, everyone involved should be familiar with the latest laws and regulations that are always changing, and each institution needs to support an appropriate number of personnel including AVs.

**Table 9:** Opinions and suggestions of survey participants about postapproval monitoring*

Statement of recommendation	Number of respondents
**Understanding and regular training of relevant laws**	**5**
**Recruitment of professional personnel including attending Vet**	**3**
Compulsory implementation of non-implemented institutions	1
Improvement of operating system including IT infrastructure	1
Listening to the employees’ comments and suggestions	1

*Request descriptive answers

## Discussion

In this study, it was confirmed that both researchers and institutions recognized the necessity of the PAM program. However, the perceived importance of the PAM varies across institutions. For institutions that show a somewhat passive attitude, it may be necessary to strengthen education to improve awareness.

A document review involves IACUC members reviewing protocols and administrative matters at committee meetings, without direct observation of experimental procedures. In contrast, procedural observation involves trained experts directly observing animal experiments to verify protocol compliance. This method ensures that all procedures are conducted ethically and according to established guidelines, providing an additional layer of oversight and accountability in research practices. While this method facilitates more precise monitoring, it requires substantial resource allocation and staffing. In crisis scenarios such as pandemics, where resources and personnel are constrained, PAM activities are frequently restricted to protocol assessments and administrative reviews. Nevertheless, exclusive dependence on this approach presents substantial constraints regarding protocol compliance verification and the efficacious acquisition of essential data [18]. This limitation is also closely related to the strategy of prioritizing high-risk studies for intensive monitoring rather than applying PAM to all experiments. To address these limitations, some institutions have implemented remote approaches to PAM, leading to an increased use of online monitoring during the pandemic, and the necessity of adapting online monitoring methods in rapidly changing laboratory environments, such as during the pandemic [9]. However, researchers’ evaluations of this approach were divided into positive and negative perspectives. This aligns with the need for a more flexible IACUC operational framework and suggests the importance of establishing a more adaptable hybrid monitoring system for future PAM implementation. The IACUC may, at its discretion, determine the best means of conducting an evaluation of the institutions’ programs and facilities [19]. The National Institutes of Health, in response to the pandemic in 2020, issued a Request for Information on Flexibilities for Conducting Semiannual Animal Facility Inspections (NOT-OD-20-145), proposing the use of remote methods for certain areas in facilities [20]. For instance, a qualified individual, as determined by the IACUC, may provide prerecorded or real-time virtual tours to the IACUC members for areas housing non-Animal Welfare Act-regulated species. This approach allows for effective inspections while minimizing physical presence during challenging circumstances. The hybrid PAM model, integrating both indirect and direct activities, offers a practical alternative that bridges the gap between these approaches. Such a model is particularly useful in emergency situations or settings with limited resources, where efficient utilization of available assets is essential.

This investigation revealed that manpower deficiencies constitute the primary impediment in conducting PAM effectively. Despite the designation of personnel for PAM in many institutions, a substantial proportion still lack specialized staff for PAM and related IACUC activities [4]. This discrepancy in PAM implementation among institutions may be due to differing perceptions—some institutions recognize PAM as an independent role whereas others consider it an additional responsibility assigned to existing personnel. These variations correlate with organizational scale and institutional leadership, indicating the need to reassess workforce distribution and roles. For smaller institutions, a potential solution is to implement centralized oversight at the regional or national level, which serves multiple institutions.

## Conclusions

This study examined the current status of the PAM in Korea and surveyed relevant professionals, confirming that additional personnel support is necessary for institutions to properly implement PAM activities as stipulated by law. Furthermore, while remote PAM activities may be permitted in disaster situations such as a pandemic, onsite visits and procedural observations are essential for more thorough and effective PAM implementation. Therefore, the future development of the PAM should focus on establishing a collaborative framework between IACUCs and institutions, which requires systematic operations through expanded administrative support and enhanced researcher training. Specifically, strategies such as a hybrid PAM model that combines refined online remote monitoring with onsite monitoring or the shared use of regional PAM infrastructure could serve as viable solutions to enhance both the efficiency and effectiveness of PAM.

## Supplementary Information

Below is the link to the electronic supplementary material.

Supplementary Material 1

## Data Availability

Data of the study may be available upon reasonable request to the corresponding authors.

## Abbreviations

AV: attending veterinarian
IACUC: Institutional Animal Care and Use Committee
PAM: Post-approval monitoring
