# Supplementary material for: The status of postapproval monitoring operation by the Institutional Animal Care and Use Committee in Korea
Source: Lab Anim Res. 2025 Oct 15;41:27. doi: 10.1186/s42826-025-00258-2 (PMC12522620; doi:10.1186/s42826-025-00258-2)
Supplement: Supplementary file 1 — Supplementary Material 1 [file 42826_2025_258_MOESM1_ESM.docx]

***Additional file 1***

***Table A1.*** *PAM activity classification table*

|  | PAM Method |
| --- | --- |
| Observation-based (Direct method) | Laboratory inspections  Veterinary or IACUC observation of selected procedures  Observation of animals by animal care, veterinary, and IACUC staff  External regulatory inspections and assessments (accompanied on site)  Examination of surgical areas, anesthetic equipment  Use of appropriate aseptic technique  Handling and use of controlled substances (confirmed on site)  Observation of laboratory practices and comparison with approved protocols |
| Document-based (Indirect method) | Continuing protocol review  Review of protocol-related health and safety issues  Review of anesthetic and surgical records  Regular review of adverse or unexpected experimental outcomes |
